# Pneumonia detection based on RSNA dataset and anchor-free deep learning detector

**DOI:** 10.1038/s41598-024-52156-7

**Published:** 2024-01-22

**Authors:** Linghua Wu, Jing Zhang, Yilin Wang, Rong Ding, Yueqin Cao, Guiqin Liu, Changsheng Liufu, Baowei Xie, Shanping Kang, Rui Liu, Wenle Li, Furen Guan

**Affiliations:** 1 Internal Medicine Department, Taizhou Fifth People’s Hospital, Taizhou, China; 2 Respiratory and Critical Care Medicine, Taizhou Fourth People’s Hospital, Taizhou, China; 3 Department of Gerontology, Dongguan First Hospital Affiliated to Guangdong Medical University, Dongguan, China; 4 Department of Gerontolog, Ruikang Hospital Affiliated to Guangxi University of Chinese Medicine, Nanning, China; 5 Respiratory and Critical Care Medicine, Ruikang Hospital Affiliated to Guangxi University of Chinese Medicine, Nanning, China; 6 State Key Laboratory of Molecular Vaccinology and Molecular Diagnostics and Center for Molecular Imaging and Translational Medicine, School of Public Health, Xiamen University, Xiamen, China; 7 Emergency Department, Zhuhai Hospital of Integrated Chinese and Western Medicine, Zhuhai, China

**Keywords:** Computational biology and bioinformatics, Medical research, Risk factors

## Abstract

Pneumonia is a highly lethal disease, and research on its treatment and early screening tools has received extensive attention from researchers. Due to the maturity and cost reduction of chest X-ray technology, and with the development of artificial intelligence technology, pneumonia identification based on deep learning and chest X-ray has attracted attention from all over the world. Although the feature extraction capability of deep learning is strong, existing deep learning object detection frameworks are based on pre-defined anchors, which require a lot of tuning and experience to guarantee their excellent results in the face of new applications or data. To avoid the influence of anchor settings in pneumonia detection, this paper proposes an anchor-free object detection framework and RSNA dataset based on pneumonia detection. First, a data enhancement scheme is used to preprocess the chest X-ray images; second, an anchor-free object detection framework is used for pneumonia detection, which contains a feature pyramid, two-branch detection head, and focal loss. The average precision of 51.5 obtained by Intersection over Union (IoU) calculation shows that the pneumonia detection results obtained in this paper can surpass the existing classical object detection framework, providing an idea for future research and exploration.

## Introduction

Pneumonia, one of the most deadly diseases worldwide, is particularly deadly in children^1^. In 2015 alone, nearly 1 million children died from pneumonia and its complications^2^. With the popularity of chest radiography and the decreasing price of the equipment, the use of chest radiography for pneumonia detection has attracted the attention of a large number of researchers and clinicians^3^. The existing chest X-ray-based pneumonia tests require, firstly, a large amount of clinical data for learning and teaching training. Second, it requires specialized radiologists as well as clinicians to make the diagnosis. The diagnosis is based on markers, clinical manifestations, and the results of physiological tests^4,5^. Currently, with the rapid development of artificial intelligence, convolutional neural networks are used in a large number of fields such as image and signal processing due to their powerful feature extraction capabilities in Euclidean space. Convolutional neural networks are equally powerful in the field of medical image and signal research^6–8^. However, based on current research, although deep learning-based object detection frameworks can achieve excellent results, they require a lot of tedious parameter settings and experience to guarantee the effectiveness of object detection frameworks because their frameworks are anchor-based^9,10^. In other words, anchor-based object detection requires manual setting of the preset bounding box used for scanning in order to achieve good object detection results. For example, we use a large preset bounding box with an approximate aspect ratio of 1:2 for lung detection with a large rectangular object, but if we use this object detection framework for lung nodule detection, we need to use a smaller square preset bounding box with an aspect ratio of 1:1. In the case of more complex when dealing with more complex subjects or setups, more complex parameter settings are required, which also places higher demands on the user^11^, which is clearly impractical and not user-friendly for clinicians without code and computer engineering experience.

Based on this, an anchor-free pneumonia detection framework based on this paper is proposed. First, the chest X-ray images undergo an image preprocessing step with denoising and image enhancement to expand the training data set, giving the network framework a better training performance. Second, the preprocessed images are fed into a feature pyramid-based anchor-free detection framework, which does not require a preset bounding box but instead a two-branch detection head, which is used to detect the centroid of the object and its scale, respectively. in addition, in order to solve the object detection. In addition, to address the class imbalance problem in object detection, focal loss^12^ is used as the loss function of the object detection framework. Finally, the final results of pneumonia detection are obtained by computing the IoU of the RSNA dataset^13^.

Our contributions can be summarized as follows.

1. A series of data pre-processing methods were used to denoise and expand the chest X-ray images, thus enhancing the training of the object detection framework;
2. An anchor-free detection framework was used for pneumonia detection, where feature pyramids, two-branch detection head, and focal loss were used;
3. The RSNA dataset is used to verify the effectiveness of the proposed framework in this paper, and the IoU experimental results demonstrate that the proposed framework can achieve good object detection results WITHOUT preset bounding box.

The remaining chapters of this paper are organized as follows: Chapter 2 gives an introduction to the methods used in this paper, Chapter 3 gives the experimental data set and the experimental results, and the last chapter presents the conclusions and the related discussion.

## Related works

With the advent of deep learning as a model with superb feature extraction capabilities on image and signal structure, the object detection task has also shifted from traditional corner detection and circle detection, for example, to deep learning-based object detection^14,15^. The initial frameworks are based on sliding windows, and this class of frameworks designs a series of sliding windows of different shapes and sizes, which are subsequently slid from the image to be measured and deep learning is used to classify each of the intercepted images. Representative works of such methods include Fast RCNN^16^ and others, also known as two-step detectors^17,18^. In addition, there is another class of one-step detectors, represented by YOLO^19^ and SSD^20^, among others. This type of framework classifies sliding windows directly, saving the time of interception before classification. In contrast, two-step detectors are generally considered to have higher classification accuracy, but their training and testing speeds are relatively slower. One-step detectors are less accurate, but due to their processing speed, they are also heavily used in real-world scenarios with higher real-time requirements.

Deep learning-based detection frameworks, although achieving excellent results and being used extensively in industrial and medical scenarios, among others^21,22^, have attracted a lot of attention in recent years. Despite the superior effectiveness of these frameworks and the possibility of choosing one-step or two-step frameworks depending on the actual needs, their framework effectiveness depends heavily on the effectiveness of the anchor^23,24^. In other words, when a trained model is used in other applications, the anchor settings need to be modified in order to obtain better results. Therefore, the effect of the anchor-based framework largely depends on the user's experience, and the framework has poor generalization performance. In the case of a large domain gap, its framework test results are not good. Based on the above, there are some novel approaches in recent years to design frameworks that discard anchors. For example, the anchor-guide framework^25^, uses guided anchors to replace the traditional complex design of anchors, thus avoiding the drawbacks associated with preset anchors. Besides, there are some novel anchor-free frameworks, including FCOS^26^ and CSP^27^, which use a point-based scheme to find the point with the highest classification accuracy and thus determine the centroid location of the object to be measured. A summary of related works is shown in Table 1.

**Table 1:** A summary of related works.

Method	Use anchor?	One/two-step
Fast RCNN^16^	Y	Two
YOLO^19^	Y	One
SSD^20^	Y	One
Guide anchor^25^	N	Two
FCOS^26^	N	One
CSP^27^	N	One

## Method

### Overall framework

**Figure 1:**
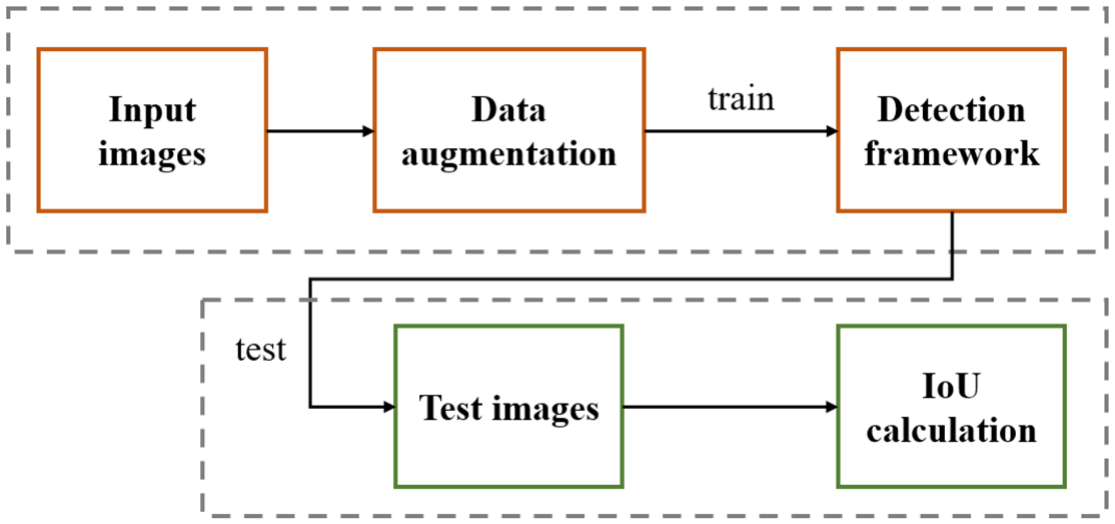
Overall flow chart of the framework.

The overall framework used in this paper is shown in Fig. 1. Firstly, the input images are first subjected to a series of data augmentation, which aims to expand the dataset through this scheme, thus improving the training effect and reducing overfitting. Secondly, the data-expanded input images are fed into the detection framework for training. The detection framework is an anchor-free detector, which does not require a preset anchor to scan the image to be measured, thus making its detection effect independent of the parameters and settings of the anchor, etc., and achieving more robust results. After training, the framework detects the image to be measured so that the IoU results are calculated and the final detection results are obtained.

### Data augmentation

The data augmentation means include a series of methods. First, the image is flipped horizontally and vertically to expand the original input image by a total of 4×. Second, the image is mirrored and then flipped both horizontally and vertically to obtain a total of 8× expansion.

In addition to the basic data augmentation, luminance augmentation and random cropping are also employed. Luminance augmentation refers to randomly changing the luminance parameter of the input image, while the luminance is randomly generated for each training, which can effectively adapt the detection framework to each type of test image and thus obtain better detection results. Random cropping means that the input image is cropped randomly, i.e., only some information about the input image is retained. Like the luminance augmentation, random cropping also makes the training input different for each training session, thus making the overall framework more robust for testing.

### Detection framework

One of the most serious problems in object detection is the inconsistent scale of the object. In the past years, only one feature extraction module has been applied in deep learning-based detectors, which is hard to detect all the objects. Specifically, if the well-trained model shows a good detection performance on big-scale objects, the detection results will greatly decrease on tiny-scale ones. Hence, a feature pyramid module is adopted to extract features with different scales, which has been verified by numerous detection frameworks.

**Figure 2:**
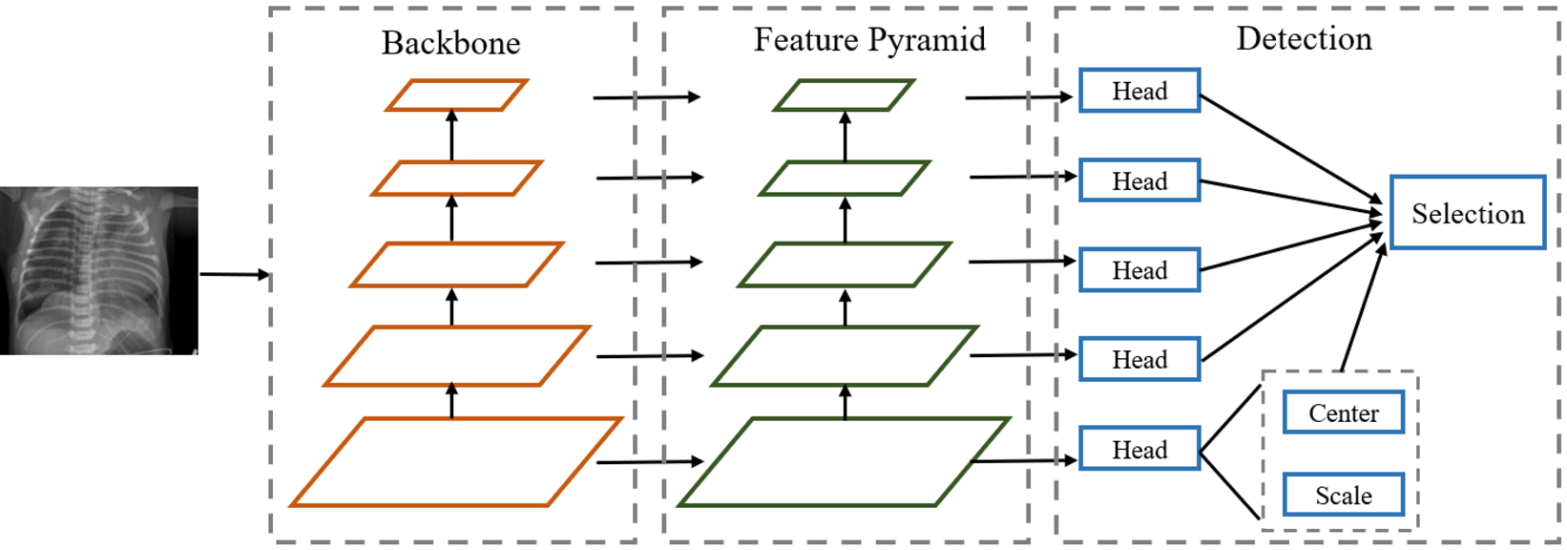
Schematic diagram of the detection framework used in this paper.

The detection framework used in this paper is shown in Fig. 2. First, an image to be measured is input to a five-layer backbone, B1 to B5, and then each layer passes through a 1 × 1 convolution kernel to form a five-layer feature pyramid, L1 to L5, where the strides of each layer are 8, 16, 32, 64, and 128, respectively. As we all know, object detection tasks include two subtasks: classification and localization. Hence, we also introduce a set of two-branch detection heads for these subtasks. In each detection head, a 3 × 3 convolutional kernel is passed first, and then two 1 × 1 convolutional kernels are passed to predict the two branches, respectively.

A series of loss functions are applied in the detection framework. One of them is for CENTER prediction, as it faces a serious class imbalance problem. In other words, since the number of CENTER points in an image is very unbalanced with the number of pixel points in the background, there are usually only a few points or even no points in a graph that belong to the CENTER category. Hence, the traditional cross-entropy loss function usually performs poorly when facing such a situation. Based on this, this paper adopts a focal loss^12^ as the loss function of the center:

1 $${L}_{center}=-\frac{1}{N}\sum_{i=1}^{W/r}{\sum }_{j=1}^{H/r}{\alpha }_{ij}{\left(1-{p}_{ij}\right)}^{\gamma }{\text{log}}\left({p}_{ij}\right)$$

where ${p}_{ij}$ is the probability of position (i, j), ranging from 0 to 1. ${\mathrm{\alpha }}_{ij}$ is the weight parameter.

In addition to center, a Smooth L1 loss has also been applied to scale predictions as follows:

2 $${L}_{scale}=\frac{1}{N}{\sum }_{n=1}^{N}{\text{Smooth}} {L}_{1}({s}_{n},g{t}_{n})$$

where N is the number of objects to be measured, s is the predicted result, and gt represents the baseline label.

Since the five detection heads train and predict the feature map separately, some uncertainty arises between each detection head. In this paper, we use a selection strategy, i.e., the largest stride in the prediction result is used as the best detection result for retention. Since the feature size is not consistent in each layer, we also set the size of the bounding boxes in each layer. Suppose the maximum distance of the regression of layer i on a certain object is ${m}_{i}$ and the regression point is (*l*, *t*, *r*, *b*). If ${m}_{i}<\left(l,t,r,b\right)<{m}_{i-1}$, then this current regression point is considered as a sample of the object. Hence, we can limit the prediction uncertainty between multiple feature layers by setting the maximum distance of each layer.

### Testing and IoU computing

Intersection over Union (IoU) is a common measure of the difference between the tested bounding box and the real box in object detection. In other words, the larger the IoU result, the better the framework is for object detection, and vice versa. The IoU is calculated as follows:

3 $$IoU=\frac{A\cap B}{A\cup B}=\frac{C}{A+B-C}$$

### Evaluation Metrics

Average precision (AP), one of the most commonly used experimental metrics for object detection, is adopted in this paper to evaluate the detection performance, which is defined as follows:

$$Average\, Precision \left(AP\right)={\int }_{r=0}^{1}p(r)dr$$

where p(r) represents the Precision-Recall curve calculated via the confusion matrix. In other words, AP is the area under the Precision-Recall curve.

Among them, AP, without special settings and explanations, defaults to the number of detected frames plus one if the test frame and the groundtruth label are considered to have overlapped at IoU > 0.5. In addition, the common AP_S_, AP_M_ and AP_L_ metrics represent the detected AP values for small, medium and large objects, respectively. Since there is no corresponding small object in the dataset used in this paper, AP_M_ and AP_L_ are used as the metrics. In addition, AR_10_, AR_M_ and AR_L_, which are measures of average Recall, are also used as the experimental metrics in this paper.

## Experimental results

### Experimental dataset

**Figure 3:**
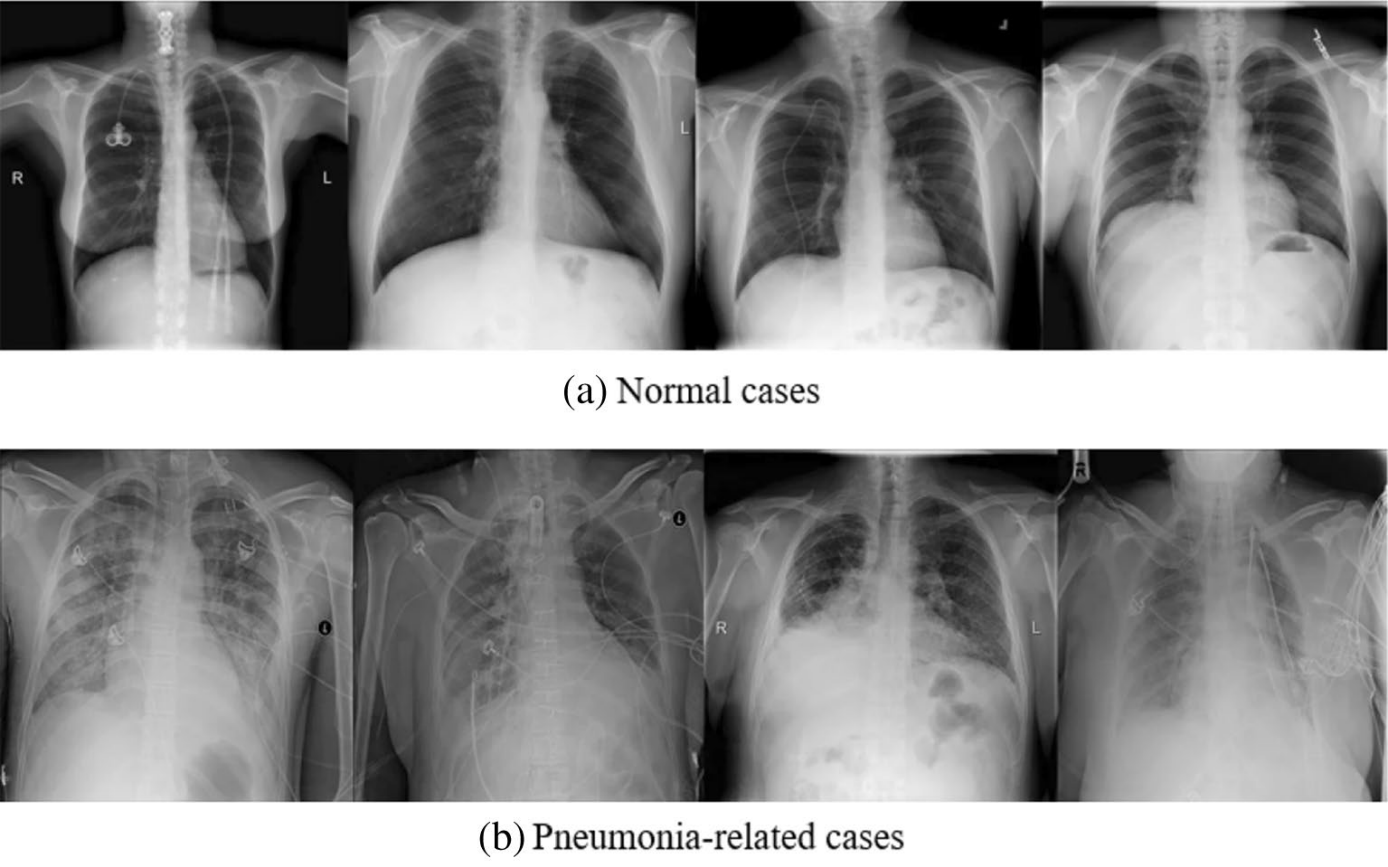
Some samples from RSNA dataset.

A dataset for pneumonia detection, RSNA^13^, was adopted as the training and testing dataset for this paper, which was published on the Kaggle platform. In this dataset, there are three different detection classifications, namely normal lung, lung without pneumonia but abnormal lung, and lung images with pneumonia. In total, 6012 different patients had at least one or more pneumonia conditions, which are collected by 18 radiologists from 16 different institutions. The mean value of labeler demographics’ experience is 10.6 years. The basic imaging examples of normal and pneumonia are shown in Fig. 3.

### Experimental setup

At the software level, ResNet-50^28^ was used to detect the backbone of the framework. The initial learning rate was set to 0.001, and the learning rate was reduced to 1/10th of the original rate after every 100 cycles with a variable learning rate setting. Anchor-free NMS was set to 0.5. ADAM^29^ was used to detect the optimizer of the framework. The settings and parameters of small, medium, and large objects follow COCO datasets^30^. The confidence score threshold for the centroid was set to 0.1. We built using the Python programming language and the Pytorch deep learning framework. The batch size was set to 1. The whole framework was experimented and accelerated on a single RTX 2080ti.

## Experimental results

The detection results of the proposed framework are shown in Table 2. Where "Ours" represents the object detection framework using only the automatically selected feature layer proposed in this paper. "Ours + DA" represents the effect of using the detection framework of this paper with data augmentation. "Ours + DA + FL" represents the result of this paper with data enhancement and focal loss function. It can be seen from the table that both improvements proposed in this paper can effectively improve the experimental metrics. In particular, the AP can be improved from 48.6 in the base framework to 50.2 and 51.5. For AP_M_ and AP_L_, the two boosts proposed in this paper can also improve their decibels by about 5% and 4%. In addition to the accuracy results, the experimental results of 96.6, 90.6 and 98.9 can be effectively achieved for the three recall results in this paper.

**Table 2:** Detection effect of the framework in this paper with ablation study.

Method	AP	AP_M_	AP_L_	AR_10_	AR_M_	AR_L_
Ours	48.6	22.4	55.1	96.1	89.6	96.3
Ours + DA	50.2	25.4	57.0	96.1	90.1	96.8
Ours + DA + FL	**51.5**	**27.6**	**58.9**	**96.6**	**92.6**	**98.9**

To better compare the experimental results of the proposed framework and other papers in this paper, a comparison is shown in Table 3. SSD, full name Single Shot MultiBox Detector^20^, is an object detection algorithm proposed by Wei Liu at ECCV 2016, which has an obvious speed advantage over Faster R-CNN and an obvious detection performance advantage over YOLO. As a one-stage detector, SSD inherits the idea of converting detection into regression from YOLO and completes object localization and classification at one time. In addition, SSD also proposes a similar Prior box based on the Anchor in Faster R-CNN, and adds a Pyramidal Feature Hierarchy based detection method, which predicts objects on feature maps with different perceptual fields.

**Table 3:** Comparison table.

Method	AP	AP_M_	AP_L_	AR_10_	AR_M_	AR_L_
SSD	25.2	10.4	30.6	74.1	52.7	86.4
Faster R-CNN	36.5	19.2	42.7	88.9	65.3	96.2
RetinaNet	45.8	25.5	52.5	**97.7**	**92.6**	**99.3**
FCOS	48.6	24.7	53.3	95.6	90.2	96.7
NYNet	49.1	22.7	57.6	94.2	88.7	96.1
Ours + DA + FL	**51.5**	**27.6**	**58.9**	96.6	**92.6**	98.9

After the design experience of R-CNN and Fast R-CNN, Ross B. Girshick proposed the new Faster RCNN in 2016^31^, which structurally has integrated feature extraction, proposal extraction, bounding box regression, and classification all integrated in one network, which makes the comprehensive performance improved. Compared with other two-stage detectors, Faster R-CNN is particularly fast in terms of detection speed. In addition, the detection accuracy is greatly improved compared to one-stage detectors.

RetinaNet^32^ is a unified object detection network consisting of a backbone and two subnetworks. the main role of the backbone is to obtain a feature map of the whole input image through a series of convolution operations. the two subnetworks perform object classification and position regression based on the output feature map, respectively. Compared with the original FPN, the convolution process of RetinaNet uses ResNet, and the upsampling and side-joining are still FPN structures. Through the backbone network, a multi-scale feature pyramid is generated. Then two sub-networks are connected at the back for classification and regression, respectively.

Most of the current advanced object detection models, such as RetinaNet, SSD, YOLOv3, and Faster R-CNN, rely on predefined anchor boxes. In contrast, FCOS^26^ is an anchor box free framework and is also proposal free, that is, it does not rely on predefined anchor boxes or proposal regions. By removing the pre-defined anchor boxes, FCOS completely avoids the complex operations on anchor boxes, such as calculating the overlap during training, and saves the memory usage during training. More importantly, all hyperparameters related to the anchor frame that are very sensitive to the final detection results are avoided in this paper. Since only non-maximum suppression (NMS) is used for post-processing, the FCOS proposed in this paper has the advantage of being simpler than previous first-order detectors based on anchor frames. In addition, an enhanced FCOS (NYNet), which is proposed by Yan et al.^33^, is adopted for comparisons. Specifically, for fair competition, the detection performance using ResNet50 is included in Table 3.

By comparing with the above classical detection frameworks, we can find that the proposed framework can achieve the best AP results. In particular, compared with SSD, Faster R-CNN, RetinaNet, and FCOS, the proposed framework achieves about 26%, 15%, 6%, and 3% higher APs, respectively. Similarly, for AP_M_ and AP_L_ , the proposed framework achieves about 17% and 28% improvement compared with SSD, which is the worst of the classical frameworks, respectively. When compared with the best classical framework, FCOS and NYNet, this framework still achieves about 3% and 2% improvement in AP, respectively. As for the AR-related metrics, since the previous classical work has achieved results close to 100, this paper can still achieve results greater than 90. Only in two items, AR_10_ and AR_L_, the comparison is about 1% lower than RetinaNet. From the table of comparison results, it can be seen that the proposed framework can effectively achieve the detection of pneumonia and achieve better results than the classical algorithms.

**Figure 4:**
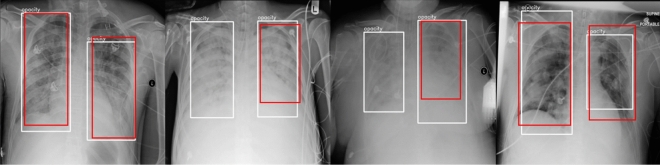
Schematic diagram of the RSNA dataset measured in practice.

The schematic diagram of the real test on the RSNA dataset is shown in Fig. 4. In the figure, the white bounding box is the groudtruth label, and the red box is the detection result of the proposed framework in this paper. From Fig. 4, it can be seen that on the first and last graphs, the framework of this paper can effectively detect the object of pneumonia, and the red and white boxes have a relatively high overlap. In both the second and third images, the proposed framework in this paper can detect the right side of the lung in place, but the detection effect is poor, probably due to the lighter color of the left image.

## Conclusion

In this paper, an anchor-free pneumonia detection framework is proposed. First, a series of data pre-processing methods are used to denoise and expand the chest X-ray images, which in turn expands the training set and improves the training effect of the detection framework. Secondly, an anchor-free detection framework incorporating feature pyramids is proposed, and ambiguity between feature layers is avoided by a selection mechanism. Finally, focal loss is used as the loss function of the framework in this paper to solve the extreme class imbalance problem in pneumonia detection. The experimental results on RSNA show that the proposed framework can effectively improve pneumonia detection. Compared with four classical object detection frameworks, including SSD, Faster R-CNN, RetinaNet, and FCOS, the proposed framework can achieve superior results, thus verifying the effectiveness and advancement of this paper's method.

## Discussion

In contrast to traditional object detection frameworks, the proposed framework does not need to be based on the anchor structure, making its effect dependent on fewer parameters to achieve object detection and localization. In addition, for some applications that require fine-tuning of parameters such as bounding box and anchor, the anchor-free framework used in this paper can also avoid these problems. In other words, for pneumonia or other medical-related object detection tasks, when the users are clinicians or medical experts, who often do not have the ability to tune the parameters for AI systems, the anchor-free framework is more friendly as an object detection framework with fewer parameters and higher applicability for such users.

Although the framework has been validated on a dataset for pneumonia detection, the effectiveness of improved schemes such as data augmentation and loss functions has also been verified. The main advantage of this anchor-free framework is that it addresses the problem that traditional detection frameworks rely too much on the parameter design of the anchor and thus design a framework that can achieve good object detection results without fine-tuning the anchor. However, the effectiveness of this anchor-free framework needs more data sets and more applications to be verified, such as pedestrian detection in autonomous driving, vehicle and ship detection in remote sensing images, and underwater object detection. In addition, the data augmentation and loss function improvement tools in this paper focus on the widespread problems in medical image object detection, including the high noise level in medical images and the small number of objects to be measured. However, whether these improvements can be applied to other medical image detection, such as tumor detection, spontaneous brain hemorrhage detection, and blood vessel detection, needs further experimental validation in future research.

## Data Availability

The dataset for this study can be requested from the RSNA Pneumonia Detection Challenge, (https://www.rsna.org/education/ai-resources-and-training/ai-image-challenge/rsna-pneumonia-detection-challenge-2018).

## References

[1] Mandell LA, Niederman MS (2019). Aspiration pneumonia. N. Engl. J. Med..

[2] Papazian L, Klompas M, Luyt C-E (2020). Ventilator-associated pneumonia in adults: a narrative review. Intensive Care Med..

[3] Hammoudi K (2021). Deep learning on chest X-ray images to detect and evaluate pneumonia cases at the era of COVID-19. J. Med. Syst..

[4] Yang F (2021). Pneumoconiosis computer aided diagnosis system based on X-rays and deep learning. BMC Med Imaging.

[5] Khatri, A., *et al.* Pneumonia identification in chest X-ray images using EMD. In *Trends in Communication, Cloud, and Big Data: Proceedings of 3rd National Conference on CCB, 2018* (Springer Singapore, 2020).

[6] Sarvamangala DR, Raghavendra VK (2022). Convolutional neural networks in medical image understanding: a survey. Evol. Intell..

[7] Xie, Y., *et al.* Cotr: Efficiently bridging cnn and transformer for 3d medical image segmentation. In *Medical Image Computing and Computer Assisted Intervention–MICCAI 2021: 24th International Conference, Strasbourg, France, September 27–October 1, 2021, Proceedings, Part III 24* (Springer International Publishing, 2021).

[8] Wu EQ (2020). Detecting Alzheimer’s dementia degree. IEEE Trans. Cogn. Dev. Syst..

[9] Zou, Z., *et al.* Object detection in 20 years: A survey. In: *Proceedings of the IEEE* (2023).

[10] Tang Z-R (2022). Multi-expert learning for fusion of pedestrian detection bounding box. Knowl. Based Syst..

[11] Guo Y (2022). Improved YOLOv4-CSP algorithm for detection of bamboo surface sliver defects with extreme aspect ratio. IEEE Access.

[12] Tran, G. S. *et al.* Improving accuracy of lung nodule classification using deep learning with focal loss. *J. Healthc. Eng.,* 2019 (2019). 10.1155/2019/5156416 PMC6378763 30863524

[13] RSNA Pneumonia Detection Challenge, https://www.rsna.org/education/ai-resources-and-training/ai-image-challenge/rsna-pneumonia-detection-challenge-2018

[14] Glumov NI, Kolomiyetz EI, Sergeyev VV (1995). Detection of objects on the image using a sliding window mode. Opt. Laser Technol..

[15] Zhang L, Zhao J, Li W (2019). Online and unsupervised anomaly detection for streaming data using an array of sliding windows and PDDs. IEEE Trans. Cybern..

[16] Girshick, R. Fast r-cnn. In *Proceedings of the IEEE International Conference on Computer Vision* (2015).

[17] Wang, X., Abhinav, S., Abhinav, G. A-fast-rcnn: Hard positive generation via adversary for object detection. In: *Proceedings of the IEEE conference on computer vision and pattern recognition* (2017).

[18] Sun X, Pengcheng W, Hoi SCH (2018). Face detection using deep learning: An improved faster RCNN approach. Neurocomputing.

[19] Redmon, J. *et al.* You only look once: Unified, real-time object detection. In *Proceedings of the IEEE conference on computer vision and pattern recognition* (2016).

[20] Liu, W. *et al.* Ssd: Single shot multibox detector. In *Computer Vision–ECCV 2016: 14th European Conference, Amsterdam, The Netherlands, October 11–14, 2016, Proceedings, Part I 14* (Springer International Publishing, 2016).

[21] McLeavy CM (2021). The future of CT: Deep learning reconstruction. Clin. Radiol..

[22] Chilamkurthy S (2018). Deep learning algorithms for detection of critical findings in head CT scans: A retrospective study. Lancet.

[23] Zhong, Y. *et al.* Anchor box optimization for object detection. In *Proceedings of the IEEE/CVF Winter Conference on Applications of Computer Vision* (2020).

[24] Kong T (2020). Foveabox: Beyound anchor-based object detection. IEEE Trans. Image Process..

[25] Wang, J., *et al.* Region proposal by guided anchoring. In *Proceedings of the IEEE/CVF Conference on Computer Vision and Pattern Recognition* (2019).

[26] Tian Z (2020). Fcos: A simple and strong anchor-free object detector. IEEE Trans. Patt. Anal. Mach. Intelli..

[27] Liu, W., *et al.* High-level semantic feature detection: A new perspective for pedestrian detection. In *Proceedings of the IEEE/CVF conference on computer vision and pattern recognition* (2019).

[28] He, K., et al. Deep residual learning for image recognition. In *Proceedings of the IEEE conference on computer vision and pattern recognition* (2016).

[29] Kingma, D. P., Jimmy, B. Adam: A method for stochastic optimization. *arXiv preprint *arXiv:1412.6980 (2014).

[30] Lin, T. Y., *et al.* Microsoft coco: Common objects in context. In *Computer Vision–ECCV 2014: 13th European Conference, Zurich, Switzerland, September 6–12, 2014, Proceedings, Part V 13* (Springer International Publishing, 2014).

[31] Ren, S., *et al.* Faster r-cnn: Towards real-time object detection with region proposal networks. *Adv. Neural Inf. Process. Syst., 28* (2015). 10.1109/TPAMI.2016.2577031 27295650

[32] Lin, T.-Y., *et al.* Focal loss for dense object detection. In *Proceedings of the IEEE international conference on computer vision* (2017).

[33] Yan N, Tao Y (2023). Pneumonia X-ray detection with anchor-free detection framework and data augmentation. Int. J. Imaging Syst. Technol..

